# Supplementary material for: The clinical effectiveness of REGEN-COV in SARS-CoV-2 infection with Omicron versus Delta variants
Source: PLoS One. 2022 Dec 2;17(12):e0278770. doi: 10.1371/journal.pone.0278770 (PMC9718412; doi:10.1371/journal.pone.0278770)
Supplement: S1 Table — (DOCX) [file pone.0278770.s001.docx]

S1 Table. Propensity Matched Cohort Characteristics by REGEN-COV Receipt.

|  | Delta Period | | Omicron Period | |
| --- | --- | --- | --- | --- |
|  | No REGEN-COV | REGEN-COV | No REGEN-COV | REGEN-COV |
| # of Patients, N (row %) | 852 | 213 | 624 | 156 |
|  |  |  |  |  |
| Demographics |  |  |  |  |
| Age, med(IQR) | 50 (38, 62) | 56 (45, 66) | 59 (48, 68) | 62 (48, 71) |
| Female | 471 (55%) | 117 (55%) | 337 (54%) | 81 (52%) |
| Race/Ethnicity |  |  |  |  |
| Non-Hispanic White | 127 (15%) | 26 (12%) | 191 (31%) | 46 (29%) |
| Hispanic White | 407 (48%) | 112 (53%) | 278 (45%) | 69 (44%) |
| Hispanic Black | 14 (1.6%) | 4 (1.9%) | 9 (1.4%) | 3 (1.9%) |
| Non-Hispanic Black | 223 (26%) | 52 (24%) | 106 (17%) | 26 (17%) |
| Other/Unknown | 81 (9.5%) | 19 (8.9%) | 40 (6.4%) | 12 (7.7%) |
| Payor |  |  |  |  |
| Commercial | 673 (79%) | 166 (78%) | 512 (82%) | 120 (77%) |
| Medicaid | 11 (1.3%) | 3 (1.4%) | 2 (0.3%) | 1 (0.6%) |
| Medicare | 63 (7.4%) | 22 (10%) | 85 (14%) | 28 (18%) |
| Unknown/Other | 105 (12%) | 22 (10%) | 25 (4.0%) | 7 (4.5%) |
|  |  |  |  |  |
| Vaccination Status |  |  |  |  |
| Vaccination Status |  |  |  |  |
| Not Vaccinated | 502 (59%) | 128 (60%) | 215 (34%) | 54 (35%) |
| Fully Vaccinated ≥ 6 Months | 76 (8.9%) | 17 (8.0%) | 206 (33%) | 54 (35%) |
| Fully Vaccinated < 6 Months | 274 (32%) | 68 (32%) | 203 (33%) | 48 (31%) |
| Boosted | 85 (10.0%) | 22 (10%) | 165 (26%) | 39 (25%) |
|  |  |  |  |  |
| Chronic Health Condition |  |  |  |  |
| History known | 636 (75%) | 175 (82%) | 496 (79%) | 128 (82%) |
| Cancer diagnosis | 92 (11%) | 46 (22%) | 109 (17%) | 36 (23%) |
| Elixhauser Comorbidities |  |  |  |  |
| AIDS | 6 (0.7%) | 4 (1.9%) | 3 (0.5%) | 1 (0.6%) |
| Alcohol Abuse | 3 (0.4%) | 1 (0.5%) | 0 (0%) | 0 (0%) |
| Deficiency Anemia | 81 ( 9.5%) | 43 (20%) | 82 (13%) | 23 (15%) |
| Rheumatoid Arthritis/CVD | 32 (3.8%) | 15 (7.0%) | 24 (3.8%) | 7 (4.5%) |
| Blood Loss Anemia | 16 (1.9%) | 11 (5.2%) | 15 (2.4%) | 5 (3.2%) |
| Congestive Heart Failure | 11 (1.3%) | 8 (3.8%) | 15 (2.4%) | 5 (3.2%) |
| Chronic Pulmonary Dis. | 69 (8.1%) | 26 (12%) | 80 (13%) | 24 (15%) |
| Coagulopathy | 30 (3.5%) | 21 (9.9%) | 25 (4.0%) | 10 (6.4%) |
| Depression | 33 (3.9%) | 16 (7.5%) | 32 (5.1%) | 9 (5.8%) |
| Uncomplicated Diabetes | 58 (6.8%) | 22 (10%) | 46 (7.4%) | 11 (7.1%) |
| Complicated Diabetes | 39 (4.6%) | 12 (5.6%) | 38 (6.1%) | 9 (5.8%) |
| Drug Abuse | 6 (0.7%) | 1 (0.5%) | 9 (1.4%) | 2 (1.3%) |
| Hypertension | 158 (19%) | 63 (30% | 154 (25%) | 43 (28%) |
| Hypothyroidism | 44 (5.2%) | 17 (9.0%) | 41 (6.6%) | 10 (6.4%) |
| Liver Disease | 46 (5.4%) | 18 (8.5%) | 45 (7.2%) | 10 (6.4%) |
| Lymphoma | 15 (1.8%) | 13 (6.1%) | 17 (2.7%) | 10 (6.4%) |
| Fluid & Electrolyte Dis. | 64 (7.5%) | 32 (15%) | 65 (10%) | 22 (14%) |
| Metastatic Cancer | 31 (3.6%) | 17 (8.0%) | 24 (3.8%) | 8 (5.1%) |
| Other Neurologic Dis. | 40 (4.7%) | 22 (10%) | 52 (8.3%) | 18 (12%) |
| Obesity | 121 (14%) | 37 (17%) | 99 (16%) | 24 (15%) |
| Paralysis | 1 (0.1%) | 3 (1.4%) | 0 (0%) | 0 (0%) |
| Peripheral Vascular Dis. | 23 (2.7%) | 12 (5.6%) | 18 (2.9%) | 5 (3.2%) |
| Psychoses | 10 (1.2%) | 7 (3.3%) | 0 (0%) | 0 (0%) |
| Pulmonary Circulation Dis. | 5 (0.6%) | 8 (3.8%) | 6 (1.0%) | 2 (1.3%) |
| Renal Failure | 24 (2.8%) | 13 (6.1%) | 20 (3.2%) | 6 (3.8%) |
| Solid Tumor, No Metastasis | 87 (10%) | 27 (13%) | 90 (14%) | 28 (18%) |
| Peptic Ulcer Disease | 3 (0.4%) | 3 (1.4%) | 9 (1.4%) | 2 (1.3%) |
| Valvular Disease | 17 (2.0%) | 7 (3.3%) | 13 (2.1%) | 4 (2.6%) |
| Weight Loss | 34 (4.0%) | 19 (8.9%) | 27 (4.3%) | 8 (5.1%) |
|  |  |  |  |  |
| Outcomes |  |  |  |  |
| Hospitalized within 30d | 22 (2.6%) | 11 (5.2%) | 19 (3.0%) | 10 (6.4%) |

AIDS: acquired immune deficiency syndrome; CVD: collagen vascular disease; d: days; Dis.: disease; IQR: interquartile range; med: median
