# Supplementary material for: The clinical effectiveness of REGEN-COV in SARS-CoV-2 infection with Omicron versus Delta variants
Source: PLoS One. 2022 Dec 2;17(12):e0278770. doi: 10.1371/journal.pone.0278770 (PMC9718412; doi:10.1371/journal.pone.0278770)
Supplement: S2 Table — (DOCX) [file pone.0278770.s002.docx]

S2 Table. Characteristics of Patients who Received REGEN-COV by Time Period.

|  | Full Cohort | Delta Period | Omicron Period | p-value |
| --- | --- | --- | --- | --- |
| # of Patients, N (row %) | 369 | 213 (57.7%) | 156 (42.3%) |  |
|  |  |  |  |  |
| Demographics |  |  |  |  |
| Age, med(IQR) | 59 (46, 70) | 56 (45, 66) | 62 (48, 71) | 0.006 |
| Female | 198 (54%) | 117 (55%) | 81 (52%) |  |
| Race/Ethnicity |  |  |  | 0.001 |
| Non-Hispanic White | 72 (20%) | 26 (12%) | 46 (29%) |  |
| Hispanic White | 181 (49%) | 112 (53%) | 69 (44%) |  |
| Hispanic Black | 7 (1.9%) | 4 (1.9%) | 3 (1.9%) |  |
| Non-Hispanic Black | 78 (21%) | 52 (24%) | 26 (17%) |  |
| Other/Unknown | 31 (8.4%) | 19 (8.9%) | 12 (7.7%) |  |
| Payor |  |  |  | 0.032 |
| Commercial | 286 (78%) | 166 (78%) | 120 (77%) |  |
| Medicaid | 4 (1.1%) | 3 (1.4%) | 1 (0.6%) |  |
| Medicare | 50 (14%) | 22 (10%) | 28 (18%) |  |
| Unknown/Other | 29 (7.9%) | 22 (10%) | 7 (4.5%) |  |
|  |  |  |  |  |
| Vaccination Status |  |  |  |  |
| Vaccination Status |  |  |  | <0.001 |
| Not Vaccinated | 182 (49%) | 128 (60%) | 54 (35%) |  |
| Fully Vaccinated ≥ 6 Months | 71 (19%) | 17 (8.0%) | 54 (35%) |  |
| Fully Vaccinated < 6 Months | 116 (31%) | 68 (32%) | 48 (31%) |  |
| Boosted | 61 (17%) | 22 (10%) | 39 (25%) | <0.001 |
|  |  |  |  |  |
| Chronic Health Condition |  |  |  |  |
| History known | 303 (82%) | 175 (82%) | 128 (82%) | >0.99 |
| Cancer diagnosis | 82 (22%) | 46 (22%) | 36 (23%) | 0.83 |
| Any Elixhauser Comorbidity | 247 (67%) | 144 (68%) | 103 (66%) | 0.84 |
| # of Elixhauser Comorbidities, med(IQR) | 1 (0, 3) | 2 (0, 4) | (1 (0, 3) | <0.001 |
| Visited UHealth within prior 1 year | 320 (87%) | 192 (90%) | 128 (82%) | 0.035 |
|  |  |  |  |  |
| Outcomes |  |  |  |  |
| Hospitalized within 30d | 21 (5.7%) | 11 (5.2%) | 10 (6.4%) | 0.78 |
| Hospitalized within 15d | 14 (3.8%) | 7 (3.3%) | 7 (4.5%) | 0.75 |
| ED Presentation within 30d | 27 (7.3%) | 16 (7.5%) | 11 (7.1%) | >0.99 |
| ED Presentation within 15d | 24 (6.5%) | 14 (6.6%) | 10 (6.4%) | >0.99 |

d: days; ED: emergency department; IQR: interquartile range; med: median; UHealth: University of Miami Hospital and Clinics Health System
