# Supplementary material for: The clinical effectiveness of REGEN-COV in SARS-CoV-2 infection with Omicron versus Delta variants
Source: PLoS One. 2022 Dec 2;17(12):e0278770. doi: 10.1371/journal.pone.0278770 (PMC9718412; doi:10.1371/journal.pone.0278770)
Supplement: Attachment — Submitted filename: responses to reviewers, rev2.docx [file pone.0278770.s004.docx]

We thank the reviewers and editors again for their thorough review of our work. Please see below for a point-by-point response to the remaining concerns.

*Reviewer #1: I appreciate the answers the authors have given. Despite the missing data and the difficulties to satisfy the request of the reviewers I think this work could be now available to be published. Maybe it could be better to avoid to infere statistical data about efficacy or not given the missing data. I think it would be more appropriate just to describe the experience.*

Thank you for your comments. We agree that data missingness is a limitation but hope we have appropriately described it such that readers can correctly interpret our findings.

*Reviewer #2: Overall, Dr. Gershengorn and colleagues provide a robust retrospective analysis of their clinical experiences with REGN-CoV Mab therapy across the respective Delta and Omicron periods at their institution. Within the limits of a retrospective observational study, they do a rigorous job of controlling for potential confounding to analyze for any differential effectiveness. The authors have helpfully clarified several important aspects in the methods about the use of any other outpatient COVID-19 therapeutics. They have also added additional important comments in the limitations section of their discussion.*

Thank you.

*A few additional comments that I would add with regards to the paper are as follows:
1. I still think that the most likely explanation for the findings of no differential effectiveness is due to inadequate power to detect a difference due to a lower number of hospitalizations and ED visits, particularly in the Omicron period. The authors adequately discuss and explore this limitation in their discussion; however, I think that at least a passing comment in the abstract is also appropriate since some people will not read the full paper. So a statement such as the following in the abstract conclusion would be useful: "Within the limitations of this study's power to detect a difference, we identified no differential effectiveness..."*

We appreciate this concern and have now amended the Abstract’s conclusions to include the proposed phrase.

*2. I would remove the final sentence of the conclusion (lines 245-247) as I don't think that it is warranted based on the available data. While it is generally true that having rigorous RCTs is always preferable to relying on in vitro or clinical data, the reality in the COVID-19 pandemic is that the rapid emergence and evolution over time of clinical variants has rendered it impractical to conduct new adequately powered RCTs for each new emergent COVID-19 variant Therefore, the FDA has had to rely on this type of indirect data to determine the EUA status and recommendations for Mab therapy use. The final sentence seems to imply that based on this data we should have continued to use REGEN-CoV until we have RCT trial data to suggest otherwise; however, there is enough uncertainty and limitations to this data that I don't think that position can be fully supported in the face of consistent and compelling in vitro data showing a loss of activity against the newer Omicron lineage variants. I would just end the conclusion with the next to last sentence saying something similar to the abstract: "Within the limitations of this study's power to detect a difference, we found no clear reduction in the apparent clinical effectiveness..."*

We agree that it is worth restating the potential limitation introduced by our sample size in the concluding paragraph and have, thus, revised the first sentence as suggested (same as in the Abstract) to do just this.

As for the last sentence, it was not our intention to suggest that clinical trials, per se, be done; but, rather, that evaluations in humans (vs in vitro or pre-clinical in vivo work) is important. As such, our choice of wording (“clinical trials”) was in error, and we’ve now changed it to “in-human evaluations”. Similarly, it was not our intent to state that REGEN-COV itself should be studied in an in-human evaluation at this point; rather, our goal was to point out the value of our study in calling attention to the fact that pre-clinical and clinical studies often have divergent findings. To try to make clearer that this comment was not intended to apply to only REGEN-COV per se, we have added the phrase “for new therapies”. With these changes, we have elected to leave the last sentence in the paper as we think this making point—the importance of studying humans for therapies to be used on humans—is valuable. We hope these changes make the sentence more appropriate (palatable?) from the perspective of the reviewer.

*3. I agree with the authors that their new 4-panel version of Figure 1 adequately addresses the prior concerns.*

Thank you.
